# Suppression of Glucosylceramide Synthase Reverses Drug Resistance in Cancer Cells Harboring Homozygous p53 Mutants

**DOI:** 10.3390/ijms27073237

**Published:** 2026-04-02

**Authors:** Md Saqline Mostaq, Mohammad N. Amin, Amanda Raphael, Celine Asbury, Anish Gupta, Xin Gu, Xianlin Han, Davorka Sekulic, Pawel Michalak, Lin Kang, Yong-Yu Liu

**Affiliations:** 1 School of Basic Pharmaceutical and Toxicological Sciences, College of Pharmacy, University of Louisiana at Monroe, Monroe, LA 71201, USA; saqlinemostaq@gmail.com (M.S.M.); aminmn@warhawks.ulm.edu (M.N.A.); aeraph7@gmail.com (A.R.); 2 Division of Biomedical Affairs and Research, Edward Via College of Osteopathic Medicine, Monroe, LA 71203, USA; asburyceline@gmail.com (C.A.); agupta.res@gmail.com (A.G.); pmichalak@ulm.vcom.edu (P.M.); 3 Department of Pathology, Louisiana State University Health Sciences Center, Shreveport, LA 70112, USA; xin.gu@lsuhs.edu; 4 Barshop Institute for Longevity and Aging Studies, University of Texas Health Science Center, San Antonio, TX 78229, USA; hanx@uthscsa.edu; 5 Global Medical Affairs, RD Hematology, Sanofi, Cambridge, MA 02139, USA; davorka.sekulic@sanofi.com; 6 Center for One Health Research, VA-MD College of Veterinary Medicine, Virginia Tech, Blacksburg, VA 24061, USA; 7 Institute of Evolution, University of Haifa, Haifa 3498838, Israel

**Keywords:** glucosylceramide synthase, p53 tumor suppressor, missense mutation, *N*^6^-methyladenosine, RNA modification, cancer stem cells, drug resistance

## Abstract

Glucosylceramide synthase (GCS) catalyzes ceramide glycosylation in response to cell stress that produces glucosylceramide and other glycosphingolipids. GCS overexpression is a cause of drug resistance and enriches cancer stem cells (CSCs) during cancer chemotherapy. Previous studies showed that GCS modulates the expression of p53 mutants and oncogenic gain-of-function (GOF) in heterozygous knock-in cell models (*TP53* R273H^−/+^). However, it is unclear whether GCS can modulate the effects of homozygous p53 mutations, which are common in many cancer cases. We report herewith that inhibition of GCS, via *UGCG* knockout and using an inhibitor (Genz-161), effectively re-sensitizes drug resistance and diminishes CSCs in colon cancer cells carrying the homozygous p53 R273H mutation. In aggressive WiDr cells carrying *TP53* R273H mutation, knockout of *UGCG* gene using CRISPR/Cas9 editing or inhibition of GCS with Genz-161 sensitized cancer cells to oxaliplatin, irinotecan and paclitaxel. With decreased ceramide glycosylation in lipidomic profiling, both *UGCG* knockout and Genz-161 treatments substantially decreased wound healing, and diminished CSCs and tumor growth under chemotherapy. Interestingly, inhibition of RNA m^6^A methylation by neplanocin A markedly increased p53 function and reversed drug resistance. Mechanistic investigation revealed that GCS inhibition downregulated methyltransferase-like 3 (METTL3) expression and decreased RNA-m^6^A modification on mutant p53 R273H effects. Altogether, our findings demonstrate that ceramide glycosylation promotes METTL3 expression and RNA m^6^A methylation in response to drug-induced stress, thereby promoting mutant p53 expression and associated GOF. Conversely, inhibition of GCS can diminish CSCs and drug resistance via reduction in m^6^A modification and advance of p53-assocaited tumor suppressive function. GCS inhibition is an achievable approach for mutant cancer treatment.

## 1. Introduction

Ceramide glycosylation catalyzed by glucosylceramide synthase (GCS) modulates cell processes in response to stress, including exposure to various therapeutic agents for cancer treatments [1,2]. GCS is a rate-limiting enzyme for glycosphingolipid (GSL) synthesis, catalyzing ceramide (Cer) glycosylation that converts Cer into glucosylceramide (GlcCer) and provides a precursor for various GlcCer-based glycosphingolipids (GSLs) [3,4]. In addition to other activities, GSLs associated with membrane proteins in GSL-enriched microdomain (GEM) modulate the cellular effects of these proteins, such as Src family kinases [5,6]. Previous reports indicate that globo-series GSLs, in particular globotriosylceramide (Gb3), play crucial roles in modulating the transcription of genes via cSrc and β-catenin signaling pathways [6,7,8]. Under treatments with anticancer drugs, increased Cer-glycosylation by GCS and cellular GSLs modulate GEMs so as to promote the expression of particular genes, including multidrug resistance gene (MDR1), fibroblast growth factor 2 (FGF-2) and even p53 mutants of cancer cells for avoiding drug-induced cell death [7,8,9].

Missense mutant proteins produced from the *TP53* gene lack the tumor suppressive activity of wild-type protein has and often exhibit oncogenic gain-of-function (GOF) [10,11,12]. Human *TP53* gene encodes p53 protein, an essential tumor suppressor that stabilizes the genome with respect to the propensity for neoplastic transformation in normal cells. Acting as a transcription factor with homo-tetramer, p53 promotes the expression of its responsive genes, including p21, Bax, Puma and others, whereby p53-dependent cell proliferation arrest or apoptosis is executed in response to genotoxic stress [10]. However, *TP53* mutations are frequently detected in cancers, particularly in more than 80% of metastatic cancers or recurred cancers, such as those of ovaries and colon [13,14]. Missense mutations at codons 175, 248, and 273 constitute approximately 19% of all p53 genetic alterations; thus, these codons are referred to as mutation hotspots [15,16,17]. In addition to other oncogenic effects on tumor progression, missense p53 mutants are causative of cancer drug resistance and CSCs [9,18,19]. Restoring the expression of wild-type p53 or reactivating p53 function has been shown to re-sensitize cancer cells carrying *TP53* mutations to anticancer treatments [11,20,21,22].

Cell stress upon treatments with anticancer drugs can enhance Cer-glycosylation and upregulate the expression of p53 mutants [6,11,12,23]. Previous studies show that the inhibition of Cer-glycosylation catalyzed by GCS can reverse drug resistance and decrease the CSC population in cancer cells with heterozygous *TP53* R273H (*TP53* R273H^+/−^) [6,9,11]. Homozygous p53 mutants are more commonly detected in cancer cases and are key players in cancer progression. To understand whether Cer glycosylation by GCS is associated with drug resistance and CSC enrichment in cancer cells carrying homozygous p53 mutations derived pathologically, rather than through genetic editing, we studied WiDr cancer cells carrying the homozygous *TP53* R273H mutation and examined the effects of the new GCS inhibitor GENZ 667161 (Genz-161).

## 2. Results

### 2.1. GCS Inhibition Reversed Drug Resistance and Reduced Tumorigenesis in p53 Mutant Cancer Cells

**Figure 1:**
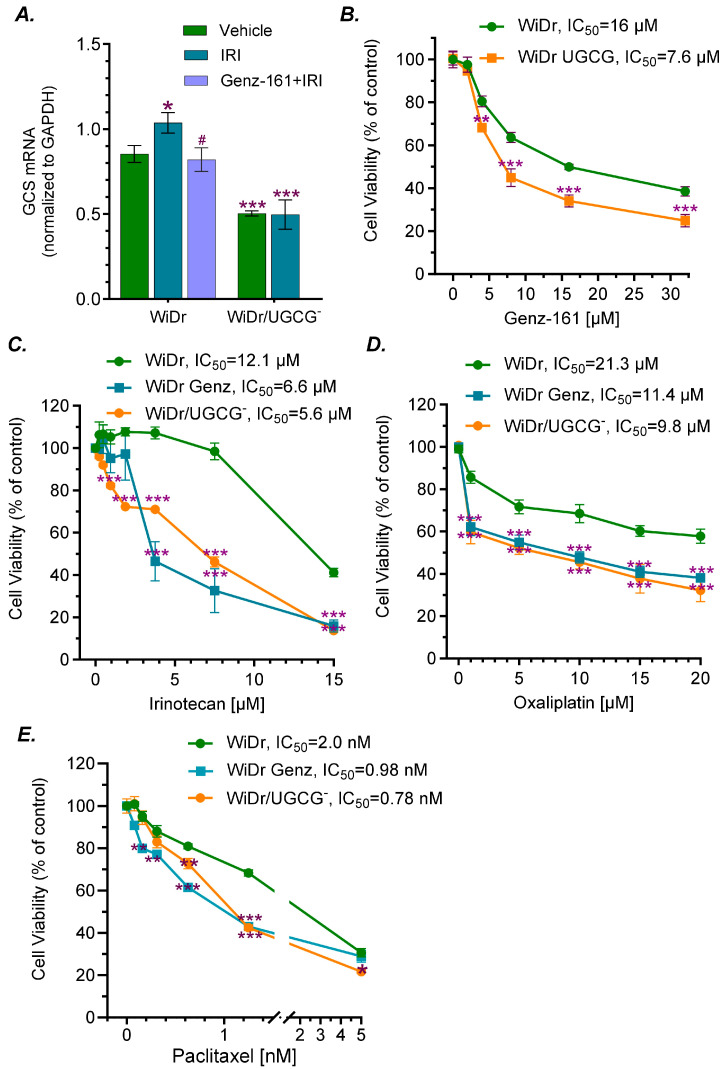
**GCS inhibition modulates drug resistance in WiDr cancer cells carrying *TP53* R273H.** (**A**) GCS mRNA levels in cancer cells. Cells were treated with 4 μM Genz-161 or vehicle for 48 h, and then exposed to irinotecan (IRI, 3 μM) for an additional 48 h. *, *p <* 0.05 compared WiDr cells treated with vehicle control. ^#^, *p* < 0.05 compared with WiDr cells treated with IRI; ***, *p* < 0.001 compared WiDr cells treated with vehicle or with IRI. (**B**) Cell response to Genz-161. Cells of WiDr and WiDr/UGCG were treated with Genz-161 or vehicle for 72 h. **, *p* < 0.01 compared with WiDr cells; ***, *p* < 0.001 compared with WiDr cells. (**C**–**E**) Cell responses to irinotecan, oxaliplatin and paclitaxel. WiDr cells were pretreated with 4 μM Genz-161 or vehicle for 48 h, and then co-treated with irinotecan or oxaliplatin and paclitaxel for an additional 72 h. **, *p* < 0.01 compared with WiDr cells treated with paclitaxel. ***, *p* < 0.001 compared with WiDr cells treated with irinotecan or other drugs alone. All these experiments were performed in replicates (n = 3 for Figure 1A; n = 6 for Figure 1B–E) and repeated at least twice. Data are expressed as mean ± SD, and significances are determined using two-way ANOVA followed by Tukey’s multiple comparisons test.

Parental WiDr cells, derived from human colorectal adenocarcinoma carrying the homozygous *TP53* R273H mutation, displayed resistance to anticancer drugs, including doxorubicin (Dox), oxaliplatin (Oxa), and 5-fluorouracil (5-Fu) [6,9,24]. Following CRISPR/Cas9-edited *UGCG* knockout and DNA sequencing confirmation, the selected WiDr/UGCG^−^ cells expressed significantly lower levels of GCS mRNA, compared with parental WiDr cells (Figure 1A). The levels of GCS mRNA were significantly decreased in WiDr/UGCG^−^ cells treated with irinotecan (IRI), compared to WiDr cells treated with IRI alone or combined with Genz-161 (Figure 1A). Assessing cell viability to GCS inhibitor Genz-161, we found that the IC_50_ values for Genz-161 in WiDr/UGCG^−^ cells are decreased by approximately 2-fold (7.6 vs. 16 μM, *p* < 0.001; Figure 1B). This suggests that UGCG may not be fully knocked out in WiDr cells. We examined cell response to several drugs that often apply to chemotherapy of colorectal cancer, and found that WiDr/UGCG^−^ cells are sensitive to these drugs tested, including irinotecan (IRI), Oxa and paclitaxel (Taxol) (Figure 1C–E). UGCG knockout significantly increased the responses of WiDr/UGCG^−^ cells to treatments of IRI, Oxa and Taxol (Figure 1C–E), and reduced the IC_50_ values by approximately 2-fold for IRI and Oxa, and by 3-fold for Taxol. Genz-161 is a potent GCS enzyme inhibitor [6], rather than an agent that decreases mRNA levels (Figure 1A). Genz-161 treatment (4 μM; ¼ of the IC_50_) re-sensitized WiDr cells and reduced the IC_50_ values for Oxa, Taxol and IRI by approximately 2-fold (Figure 1B–E). We observed that suppressing GCS moderately sensitized Taxol in WiDr cells (Figure 1E), rather than producing the remarkable effect reported in other cancer cells [25]. This difference may be associated with Taxol’s action on microtubules and colon cancer WiDr cells carrying the TP53 R273H mutation. Previous studies showed that GCS inhibition reversed Dox resistance in knock-in-p53-mutant models [11]. Present results indicate that GCS is involved in modulating multidrug resistance of homozygous *TP53* R273H cancer cells; moreover, Genz-161, a potent GCS inhibitor, can effectively re-sensitize mutant cancer cells to anticancer drugs.

**Figure 2:**
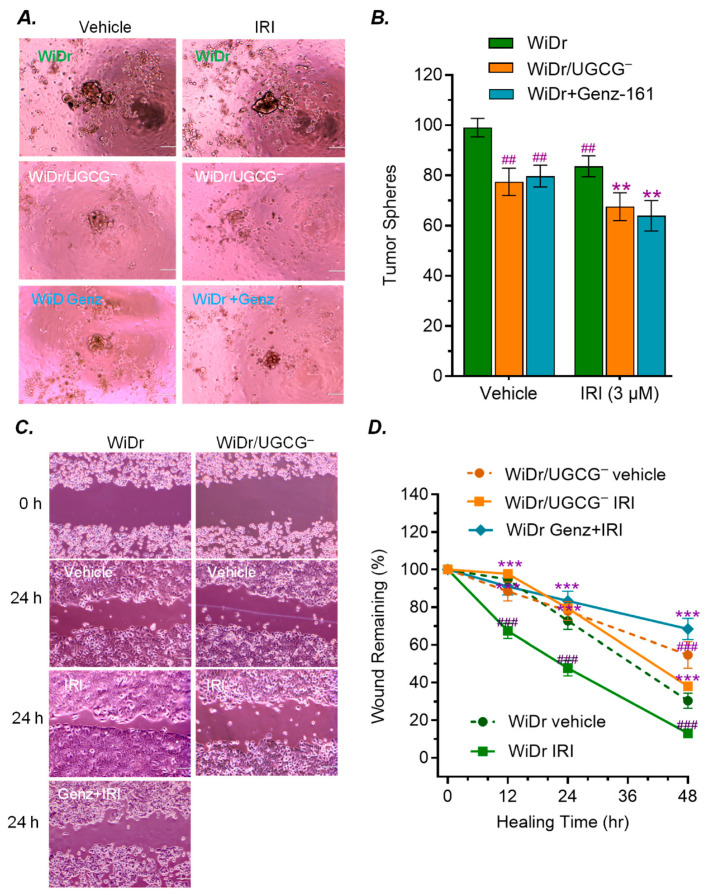
**GCS inhibition decreases tumor sphere and wound healing of WiDr cancer cells.** (**A**) Tumor sphere of WiDr cells. After pre-treatments with Genz-161 (4 μM) or vehicle, cells were planted in 5% FBS EMEM medium containing Genz-161 alone or combined with irinotecan (IRI, 3 μM) for 6 days. Images were captured with 100× magnification. Scale bar represents 100 μm. (**B**) Inhibition of GCS reduces tumor sphere formation. ^##^, *p* < 0.01 compared with WiDr cells treated with vehicle; **, *p* < 0.01 compared WiDr cells treated with IRI. (**C**) Wound healing. Cells were pretreated with Genz-161 (4 μM) or vehicle for 48 h, and then exposed to IRI (3 μM) for an additional 48 h. WiDr/UGCG cells treated with IRI alone for 48 h. Images were magnified 100×; scale bar equals to 100 μm. (**D**) GCS inhibition decreased the wound healing of WiDr cells. ^###^, *p* < 0.001 compared with WiDr cells treated with vehicle. ***, *p* < 0.001 compared with WiDr cells treated with IRI. All these experiments were performed in triplicate and repeated at least twice. Data are expressed as mean ± SD and significances are determined using two-way ANOVA followed by Tukey’s multiple comparisons test.

To further assess functional consequences of GCS inhibition, we conducted assays of tumor sphere formation and wound healing. Indeed, GCS inhibition decreased the tumorigenesis and cellular migration of WiDr cancer cells exposed to IRI. WiDr/UGCG^−^ cells formed significantly fewer and smaller tumor spheres compared to WiDr cells (approximately 70% reduction; Figure 2A,B). Genz-161 treatment displayed the same effects as UGCG knockout, significantly decreasing tumor sphere formation of WiDr cells (Figure 2A,B). IRI exposure enhanced the wound healing of WiDr cells; however, under the same conditions, wound healing was markedly decreased in both WiDr/UGCG^−^ cells and WiDr cells treated with Genz-161, compared with WiDr cells (Figure 2C,D). At 24 h and 48 h post-scratch, the wound areas in WiDr/UGCG^−^ cells remained approximately 2-fold (44% vs. 78%) and 3-fold (13% vs. 49%) larger than those in WiDr cells (Figure 2D). Genz-161 treatment displayed a similar effect on decreasing the wound healing of WiDr cells exposed to IRI.

### 2.2. GCS Inhibition Reduced the Enrichment of Cancer Stem Cells Through Modulating the Expression of TP53 R273H Mutant Proteins

**Figure 3:**
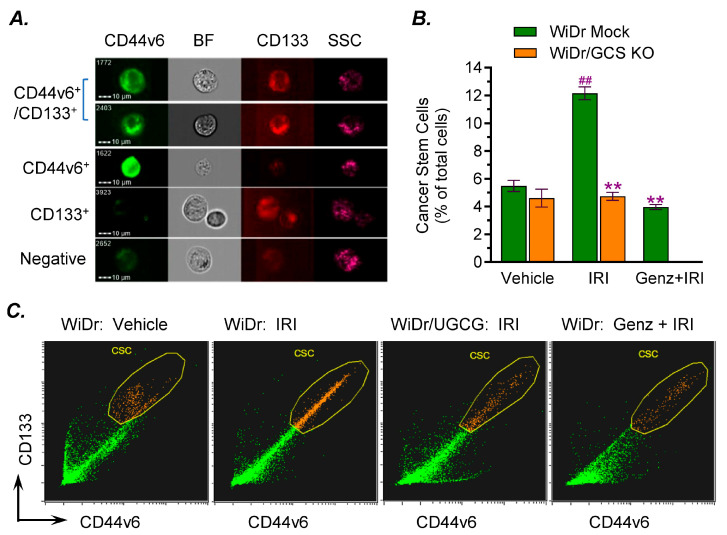
**GCS inhibition decreases cancer stem cell population of WiDr cells exposed to irinotecan.** Cells were planted in 5% FBS EMEM medium containing 3 μM IRI alone or combined with 4 μM of Genz-161 for 6 days. (**A**) Imaging flow cytometry analysis for colon CSCs (CD44v6^+^/CD133^+^). BF, bright field. (**B**) GCS inhibition decreased CSC population. **, *p* < 0.002 compared to WiDr cells treated with vehicle; ^##^, *p* < 0.0015 compared to WiDr cells treated with IRI. (**C**) Representative CSC plots of cancer cells under various treatments. All these experiments were performed in triplicate and repeated at least twice. Data are expressed as mean ± SD, and significances are determined using one-way ANOVA followed by Tukey’s multiple comparisons test.

Previous studies indicate that GCS can enrich CSCs during chemotherapy and contribute to drug resistance and metastasis [8,11,26]. Using imaging flow cytometry, we assessed cancer stem cells (CSCs) in WiDr cell lines exposed to IRI. In vehicle treatments, there is no significant difference between WiDr and WiDr/UGCG^−^ cells (Figure 3A,B). Irinotecan treatments significantly increased CSC population in WiDr cells, compared to WiDr cells treated with vehicle (Figure 3A,B). Further, we found that under irinotecan treatments, GCS inhibition significantly decreased CSC numbers of WiDr/UGCG^−^ cells or WiDr cells treated with Genz-161 by approximately 2-fold compared with WiDr cells (12.1% vs. 4.7% or 3.9%, *p* < 0.01) (Figure 3A–C). These results indicate that anticancer drugs may enrich CSCs via ceramide glycosylation by GCS. The inhibition of GCS, by either genetic manipulation or by an enzyme inhibitor, can significantly decrease CSC enrichment during drug treatments.

**Figure 4:**
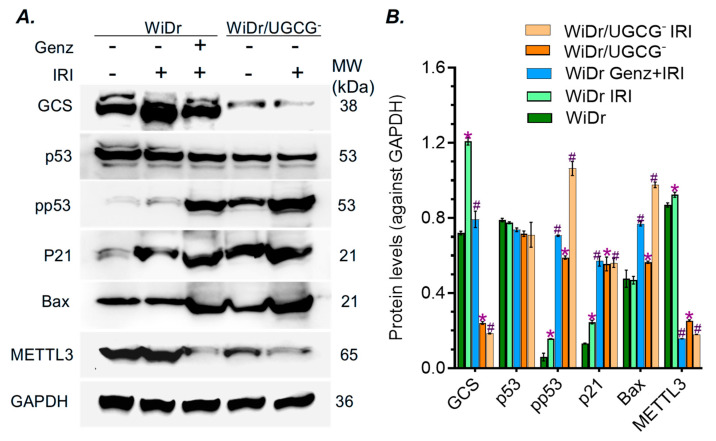
**GCS inhibition modulates the expression of p53-responsive proteins in WiDr cancer cells.** (**A**) Western blotting for protein expression. Cells were pretreated with 4 μM Genz-161 or vehicle for 48 h, and then exposed to 3 μM IRI for an additional 48 h. Equal amounts of soluble proteins (50 μg/lane) were resolved 4–12% SDS-PAGE and immunoblotted with primary antibodies and HP-conjugated protein C. (**B**) GCS modulated the expression of the p53 R273H mutant. *, *p* < 0.001 compared to WiDr cells treated with vehicle; ^#^, *p* < 0.001 compared to WiDr cells treated with IRI. All these experiments were performed in triplicate and repeated at least twice. Data are expressed as mean ± SD and significances are determined using one-way ANOVA followed by Tukey’s multiple comparisons test.

To understand how the inhibition of GCS reverses drug resistance and tumorigenesis of WiDr cells carrying the p53 mutation, we assessed the alterations of protein expression of cells under treatments. Irinotecan treatments significantly increased GCS protein levels of WiDr cells, compared with vehicle (Figure 4A,B). With the lower mRNA levels (Figure 1A), GCS protein levels were significantly decreased, by approximately 3-fold (0.24 vs. 0.72) and 6-fold (0.20 vs. 1.20, *p* < 0.001) in WiDr/UGCG^−^ cells treated with vehicle or irinotecan, compared with those of WiDr cells (Figure 4A,B). Notably, besides the canonical GCS (bottom band), an additional isoform of the protein was present in WiDr cells (Figure 4A, upper band). This may suggest that UGCG knockout silenced the canonical GCS protein, rather than its isoform or a post-translationally modified form. Genz-161 treatments did not have a significant effect on GCS protein levels of WiDr cells (Figure 4A,B). Interestingly, GCS inhibition substantially increased the levels of phosphorylated p53 (pp53, Ser15), by approximately 5-fold (0.15 vs. 0.71, *p* < 0.001) in WiDr cells treated with Genz-161 and 7-fold (0.15 vs. 1.06, *p* < 0.001) WiDr/UGCG^−^ cells treated with IRI (Figure 4A,B). Consistently, the protein levels of p53-responsive genes, such as p21 and Bax, which can induce cell proliferation arrest and apoptosis, are also significantly increased by approximately 2- to 3-fold (*p* < 0.001) in cells with GCS inhibition (Figure 4A,B). We also found that methyltransferase-like 3 (METTL3), which is reported to be responsible for RNA m^6^A methylation [9,27], was significantly decreased by GCS inhibition (~5-fold, *p* < 0.001), either in WiDr cells treated with Genz-161 and IRI or WiDr/UGCG^−^ cells treated with IRI alone (Figure 4A,B). These results clearly indicate that GCS inhibition can effectively advance pp53 expression and its dependent function in suppressing cancer stem cells and reverse drug resistance. This reactivating effect of GCS inhibition on functional p53 expression may be correlated with decreased METTL3 expression.

### 2.3. GCS Inhibition Decreased Tumorigenesis and Sensitized Chemotherapeutic Effects in Mice Bearing p53 R273H Mutant Tumors

**Figure 5:**
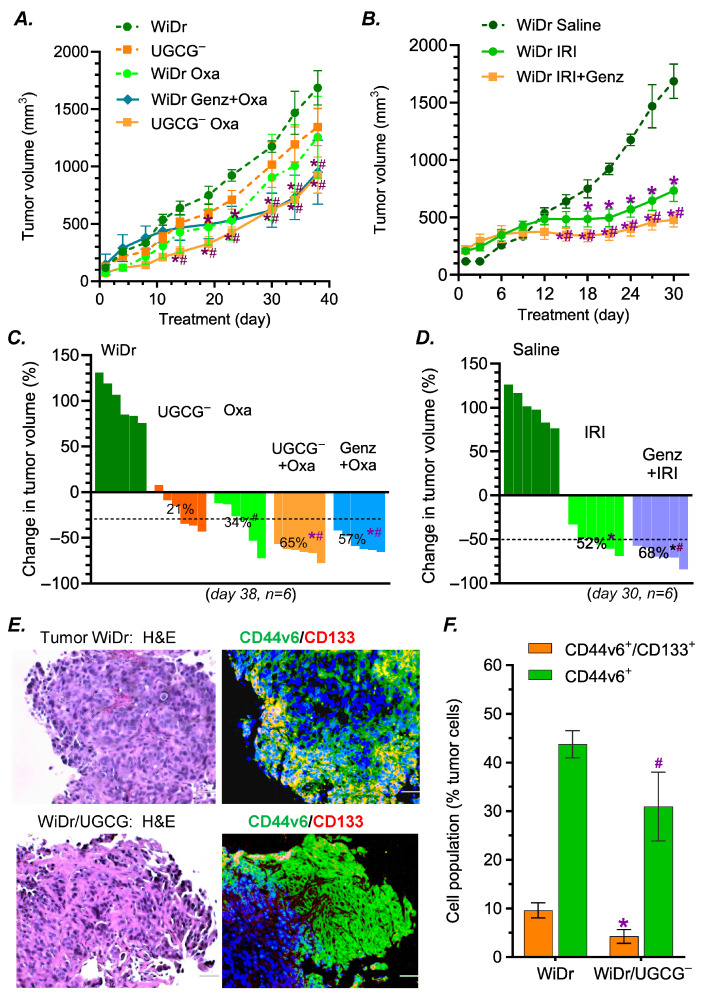
**GCS inhibition increases the effects of anticancer drugs on WiDr tumors.** (**A**) Tumor growth in mice treated with oxaliplatin. Mice bearing tumors generated from WiDr or WiDr/UGCG^−^ cells were treated with vehicle, oxaliplatin (Oxa 2 mg/kg, i.p., once every 6 days) and combination (Oxa 2 mg/kg, i.p., once every 6 days and Genz-161 4 mg/kg, i.p., once every 3 days; 6 mice/group) for 38 days. *, *p* < 0.001 compared with WiDr tumors treated with saline, and ^#^ *p* < 0.07 compared with WiDr tumors treated with Oxa. (**B**) Tumor growth in mice treated with irinotecan. Mice bearing tumors generated from WiDr cells were treated with saline, irinotecan (IRI 2 mg/kg, i.p., once every 6 days) and combination (IRI 2 mg/kg, i.p., once every 6 days and Genz-161 4 mg/kg, i.p., once every 3 days; 6 mice/group) for 30 days. *, *p* < 0.001 compared with WiDr tumors treated with saline; ^#^, *p* < 0.03 compared with tumors treated with IRI. (**C**) Waterfall plot representing the response to Oxa treatments at day 38 of treatments. *, *p* < 0.001 compared with WiDr tumors treated with saline; ^#^ *p* < 0.05 compared with WiDr tumors treated with Oxa. (**D**) Waterfall plot representing tumor response to 30 days IRI treatments. *, *p* < 0.001 compared with WiDr tumors treated with saline; ^#^ *p* < 0.05 compared with tumors treated with IRI. (**E**) H&E and immunofluorescence staining of tumors treated with Oxa. Tumor sections were stained with H&E or fluorescent antibodies for CSC markers (CD44v6/CD133). Green, Alexa Fluor 448–CD44v6; red, APC–CD133; blue, DAPI nuclear counterstain. Images were magnified 200×; scale bar represents 50 μm. Immunohistochemistry assays were performed in three tumors and repeated twice. (**F**) Colon cancer stem cells (CD44v6^+^/CD133^+^) in tumors. *, *p* < 0.001 compared with WiDr tumors treated with Oxa; ^#^, *p* < 0.01 compared with WiDr tumors treated with Oxa. Each group in the animal study including three males and three females. Data are expressed as mean ± SE and significances are determined using a two-way ANOVA followed by Tukey’s multiple comparisons test.

We examined the effects of GCS inhibition on tumorigenesis as well as drug resistance in mice bearing homozygous p53 R273H tumors. WiDr/UGCG^−^ tumors grew significantly slower and were more sensitive to Oxa and IRI treatments than WiDr tumors, consistent with decreased levels of GCS mRNA (Figure 5A–D). During days 23–38 of growth, tumor volumes of WiDr/UGCG^−^ cells were significantly decreased compared with those of WiDr. After 38 days of Oxa treatments, the volumes of WiDr/UGCG^−^ tumors (WiDr/UGCG^−^ Oxa) significantly decreased, by approximately 65%, compared with those of WiDr tumors (WiDr Oxa, Figure 5C). Interestingly, Genz-161 treatments (4 mg/kg, every 3 days) produced the same effect as UGCG knockout; these treatments sensitized WiDr tumors to Oxa, and the tumor volumes in the combination group (WiDr Genz + Oxa) decreased by approximately 57% compared with WiDr tumors treated with Oxa alone at day 38 (WiDr Oxa) (Figure 5A,C). Genz-161 also significantly sensitized WiDr tumors to IRI treatments, and the tumor volumes decreased by approximately 68% after 30 days of treatment (Figure 5B,D). These treatments, including the combination of Oxa and Genz-161, did not have a significant effect on the body weights or other tissues (liver, kidney and bone marrow) (Supplementary Figure S1).

Immunofluorescence staining showed that colon CSCs (markers CD44v6^+^/CD133^+^) of WiDr/UGCG^−^ tumors significantly decreased by approximately 2-fold (9.6% vs. 4.2% of total tumor cells, *p* < 0.001) compared with WiDr tumors treated with Oxa (Figure 5E,F). The CD44v6^+^ cells were also significantly decreased in WiDr/UGCG^−^ tumors treated with Oxa (Figure 5E,F).

### 2.4. GCS Inhibition Modulated the Expression of p53 and p53-Responsive Genes in Tumors Carrying p53 R273H Mutant

**Figure 6:**
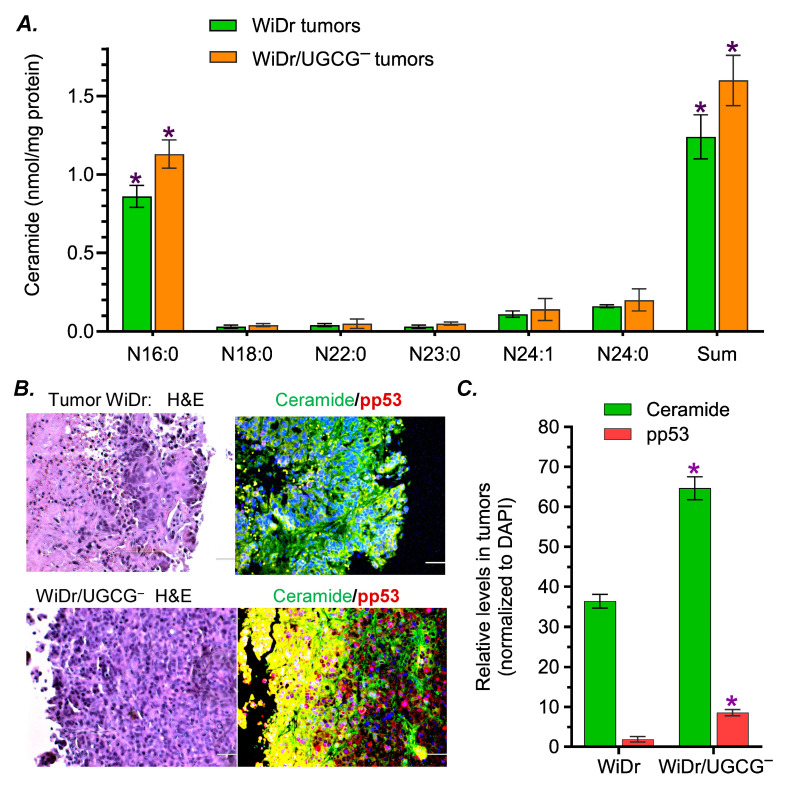
**Suppression of GCS decreases ceramides in tumors treated with oxaliplatin.** Tumor-bearing mice were treated with Oxa (2 mg/kg, i.p., once every 6 days) for 38 days. (**A**) Tumor ceramides analyzed with shotgun lipidomics. *, *p* < 0.01 compared with WiDr tumors treated with Oxa. (**B**) Immunofluorescence of tumor ceramide and pp53. Tumor sections were stained with H&E or fluorescent antibodies. Green, Alexa Fluor 488–ceramide; red, Alexa Fluor 555–pp53; blue, DAPI nuclear counterstain. Representative images were magnified 200×; scale bar represents to 50 μm. (**C**) The relative levels of tumor ceramide and pp53. *, *p* < 0.001 compared with WiDr tumors treated with Oxa. All these assays were performed in triplicate and repeated twice. Data are expressed as mean ± SD, and significances are determined using a one-way ANOVA followed by Tukey’s multiple comparisons test.

Previous studies showed that the inhibition of GCS increases cellular ceramide and decreases glucosylceramide, which can modulate mutant p53 expression [6,22,28]. We assessed and characterized the effects of GCS inhibition on the expression of p53R273H in WiDr tumors. Lipidomic analysis of tumor tissues showed that total ceramide levels in WiDr/UGCG^−^ tumors were significantly increased, with decreased cerebroside and increased sphingomyelin, compared with those of WiDr tumors treated with oxaliplatin (Figure 6A, Supplementary Figure S2). Among ceramide species, the levels of C16 ceramide (d18:1/16:0, N16:0, Figure 6A) and C16 sphingomyelin (d18:1/16:0, N16:0, Supplementary Figure S2) were remarkably enhanced in WiDr/UGCG^−^ tumors, compared to those in WiDr tumors. In immunohistochemistry assessments of tumor sections, we also found that the relative levels of tumor ceramide (stained with fluorescence-conjugated antibodies) were significantly increased by approximately 2-fold in WiDr/UGCG^−^ tumors, compared with WiDr tumors treated with Oxa (Figure 6B,C). The relative levels of nuclear pp53 proteins were substantially increased by approximately 5-fold in WiDr/UGCG^−^ tumors (1.7% vs. 8.5% of DAPI) compared with WiDr tumors treated with Oxa (Figure 6C).

**Figure 7:**
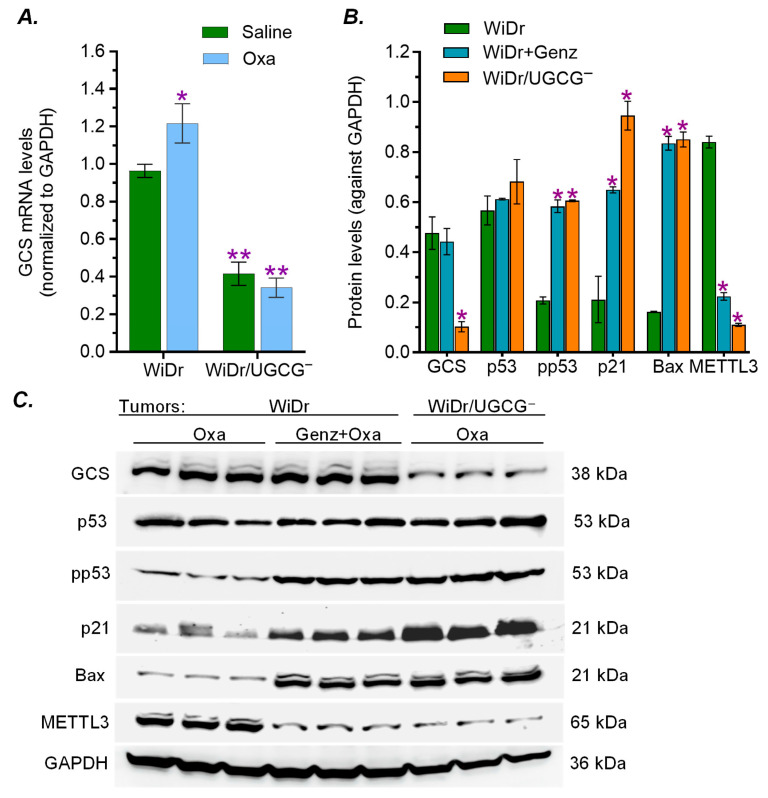
**GCS inhibition modulates expression of p53-responsive proteins in tumors treated with oxaliplatin.** Tumor-bearing mice were treated with Oxa (2 mg/kg) alone or combined with Genz-161 (Oxa 2 mg/kg and Genz-161 4 mg/kg). (**A**) GCS mRNA levels in tumors. *, *p* < 0.01 compared with WiDr tumors treated with saline; **, *p* < 0.001 compared with WiDr tumors treated with Oxa. Equal amounts of soluble proteins (50 μg/lane) were applied for Western blotting. (**B**) GCS inhibition modulated tumor protein expression. *, *p* < 0.001 compared with WiDr tumors treated with Oxa. (**C**) Western blotting of tumors. Data are expressed as mean ± SD, and significances are determined using a one-way ANOVA followed by Tukey’s multiple comparisons test.

Consistent with the cell models (Figure 4), we observed that GCS mRNA and protein levels were decreased by UGCG knockout in WiDr/UGCG^−^ tumors treated with Oxa, compared with those of WiDr tumors (Figure 7A,B). The protein levels of GCS in WiDr/UGCG^−^ tumors were decreased by approximately 5-fold (0.1 vs. 0.48, *p* < 0.001) compared to those of WiDr tumors under the same treatments (Figure 7B,C). Combination treatments (Genz-161 + Oxa) did not have any significant influence on GCS expression of WiDr tumors. GCS inhibition, by using either UGCG knockout or Genz-161 treatments, markedly increase functional p53 and p53-responsive genes to suppress tumors. The pp53 levels increased by approximately 3-fold (0.2 vs. 0.61, *p* < 0.001) in WiDr/UGCG^−^ tumors and WiDr tumors treated with Genz-161 compared with WiDr treated with Oxa alone (Figure 7B,C). Consequently, the protein levels of p21 and Bax were significantly increased by approximately 4-fold (0.21 vs. 0.94, *p* < 0.001) and 5-fold (0.16 vs. 0.85, *p* < 0.001) in WiDr/UGCG^−^ tumors compared with WiDr tumors (Figure 7B,C). Genz-161 treatments displayed similar effects in WiDr tumors treated with the combination as UGCG knockout did. METTL3 protein levels were significantly decreased by GCS inhibition, either in WiDr tumors treated with Genz-161 (4-fold, 0.23 vs. 0.81, *p* < 0.001) or WiDr/UGCG^−^ tumors (7-fold, 0.11 vs. 0.81, *p* < 0.001) compared with WiDr tumors treated with Oxa (Figure 7B,C). These results further indicate that the inhibition of GCS can markedly increase the expression of pp53 and p53-responsive genes to suppress tumorigenesis and reverse drug resistance through the modulation of RNA m^6^A methylation.

### 2.5. Ceramide Glycosylation Upregulated the Expression of METTL3 and RNA m^6^A Methylation for the Expression of p53 R273H Protein and Drug Resistance

**Figure 8:**
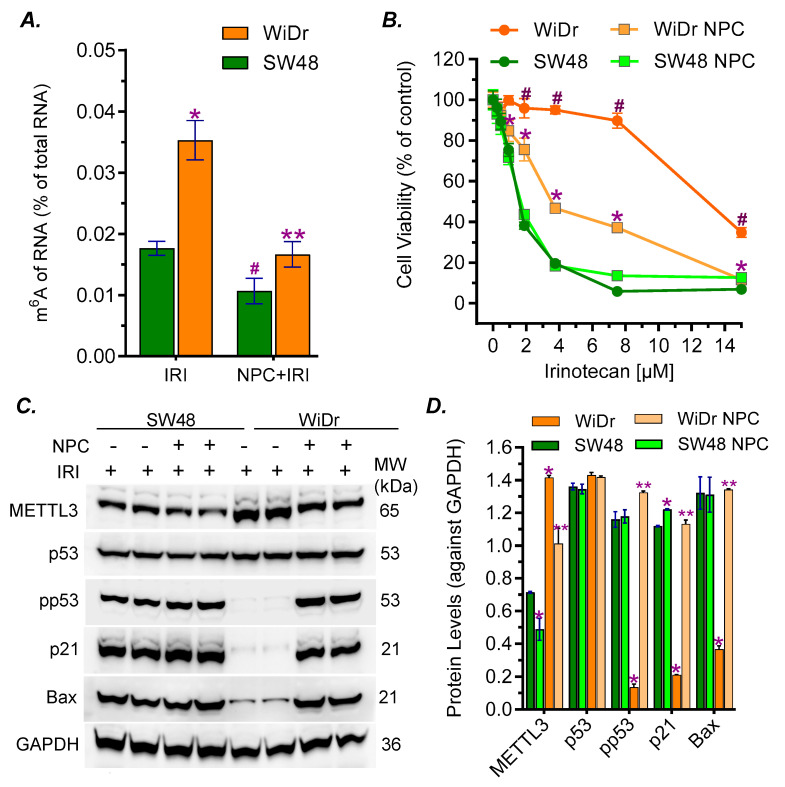
**Modulated expression of p53-responsive proteins are correlated with RNA m^6^A methylation**. (**A**) ELISA of RNA-m^6^A. Cells were pretreated with 200 nM NPC or vehicle for 48 h and co-treated with 3 μM IRI for additional 48 h. Equal amount of extracted toral RNA (200 ng/reaction) was applied to ELISA. *, *p* < 0.001 compared with SW48 cells treated with IRI; ^#^, *p* < 0.001 compared with SW48 cells treated with IRI alone; **, *p* < 0.001 compared with WiDr cell treated with IRI. (**B**) Cell response to IRI. Cells were pretreated with 200 nM NPC or vehicle for 48 h, and co-treated with increasing concentrations of IRI for additional 72 h. ^#^, *p* < 0.001 compared with SW48 cells treated with vehicle; *, *p* < 0.001 compared with WiDr cells treated with vehicle or SW48 cells pretreated with NPC. IC_50_ values: WiDr = 14.79 ± 1.08 μM, WiDr NPC = 3.1 ± 0.05 μM (*p* < 0.001 compared with WiDr treated with vehicle); SW48 = 1.45 ± 0.04 μM; SW48 NPC = 1.31 ± 0.03 μM. (**C**) Western blots of METTL3 and other proteins. Cells were pretreated with 200 nM NPC or vehicle for 48 h and co-treated with 3 μM IRI for additional 48 h. Equal amounts of soluble proteins (50 μg/lane) were applied for Western blotting. (**D**) Relative levels of p53-associated proteins. *, *p* < 0.001 compared with SW48 cells with IRI. **, *p* < 0.001 compared with WiDr cells treated with IRI. All these experiments were performed in triplicate and repeated at least twice. Data are expressed as mean ± SD and significances are determined using one-way ANOVA or two-way ANOVA followed by Tukey’s multiple comparisons test.

**Figure 9:**
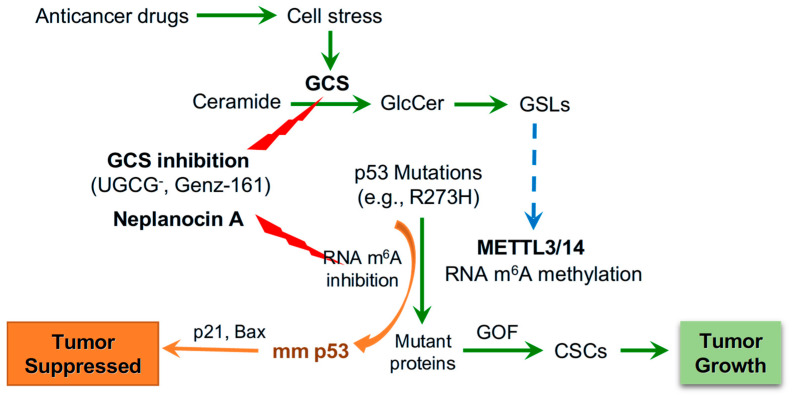
**Ceramide glycosylation by GCS modulates RNA m^6^A methylation in response to cell stress expressing mutant p53 R273H.** In response to anticancer drugs, cell stress upregulates the expression of glucosylceramide synthase (GCS), and ceramide glycosylation by cancer cells enhances the production of glucosylceramide (GlcCer) and subsequently glycosphingolipids (GSLs). GSLs induce expression of methyltransferase like-3 (METTL3) and RNA *N*^6^-methyladenosine (m^6^A) methylation of cancer cells carrying p53 mutations (*TP53* R273H). RNA m^6^A modification upregulates the expression of mutant proteins (m p53) and the gain-of-function (GOF) enriches cancer stem cells (CSCs) for tumor growth. Conversely, GCS inhibition (using either UGCG knockout or inhibitor Genz-161) or METTL3 inhibition and further RNA m^6^A modification can effectively advance p53-associated tumor suppression through the increase in functional p53 protein(s) or modified mutant p53 (mm p53).

We characterized the influence of m^6^A methylation in drug resistance of WiDr cells carrying the R273H *TP53* mutation compared to SW48 cancer cells that carry wild-type *TP53* [11,29]. Previous studies showed that neplanocin A (NPC) can inhibit RNA m^6^A methylation [9,30]. We examined the m^6^A-RNA levels of cancer cells under treatments using ELISA. Cell stress in IRI treatments greatly increased the m^6^A-RNA levels of WiDr cells, by 2-fold (0.018% vs. 0.035%, *p* < 0.001), compared with SW48 cells (Figure 8A). NPC treatments significantly decreased m^6^A-RNA levels, by 1.6-fold (0.011% vs. 0.018%, *p* < 0.001) in SW48 cell and by 2-fold (0.016% vs. 0.038%, *p* < 0.001) in WiDr cells, compared with cells exposed to IRI (Figure 8A). NPC treatments significantly sensitized WiDr cells to IRI and significantly decreased the IC_50_ values, by approximately 4-fold (3.1 μM vs. 12 μM, *p* < 0.001), compared with WiDr cells with vehicle (Figure 8B). However, NPC treatments displayed less effect on the response to IRI in SW48 cells that carry wild-type *TP53* (Figure 8B). In the Western blot analysis, we found that METTL3 protein levels were higher in WiDr cells treated with either IRI alone or the combination of IRI and NPC, by approximately 2-fold (0.71 vs. 1.4, *p* < 0.001), compared with SW48 cells under the same conditions. NPC treatments decreased the protein levels of METTL3 in both WiDr and SW48 cell lines by approximately 30% compared to those treated with IRI alone. NPC treatments remarkably increased pp53 protein levels in WiDr cells treated with combination, by approximately 10-fold (0.13 vs. 1.3, *p* < 0.001), compared with WiDr cells treated with IRI alone (Figure 8C,D). Similarly, the protein levels of p21 and Bax were also significantly increased in WiDr cells treated with NPC, by approximately 5-fold (0.21 vs. 1.13, *p* < 0.001) and 4-fold (0.37 vs. 1.34, *p* < 0.001), respectively, in WiDr cells treated with combination, compared with those treated with IRI alone (Figure 8C,D). Under the same treatment conditions, there was no significant difference between the pp53 levels of SW48 cells treated with NPC and those treated with vehicle. These results clearly indicate that RNA m^6^A methylation catalyzed by METTL3 plays a critical role in modulating the expression of functional p53 by cancer cells in response to drug-induced cell stress (Figure 9).

## 3. Discussion

Our present study indicates that GCS inhibition can reverse drug resistance and inhibit the tumor progression of aggressive cancer cells carrying the homozygous p53 R273H mutation. As a common response to cell stress induced by anticancer drugs, Cer-glycosylation by GCS increases GSLs, including globotriosylceramide Gb3 [8,31,32,33]. Overexpression of GCS and other enzymes in GSL synthesis are correlated with drug resistance and CSCs of various cancers, including colorectal cancer, triple-negative breast cancer, hepatocarcinomatous and melanoma [34,35,36]. Targeting GCS as well as GSLs (GD2, Gb3) is emerging as an effective approach for improving the outcome of chemotherapy and immunotherapy [1,2,37,38,39]. Our previous work reveals that Cer-glycosylation by GCS can modulate the protein expression of p53 mutations and the oncogenic GOF of heterozygous p53 mutant models (p53 R273H^−/+^ generated by CRISP/cas9 editing) [6,9,11]. Our present studies demonstrate that the inhibition of GCS, by either UGCG knockout or a GCS inhibitor, can reverse cancer drug resistance and eliminate CSC enrichment of homozygous p53 R273H cancer cells. WiDr cells, derived from colon adenocarcinoma carrying the p53 R273H mutation, are notorious for drug resistance and aggressive growth [6,40,41]. In the present study, we see knockout UGCG gene that encodes GCS greatly re-sensitized WiDr/UGCG^−^ cells to several anticancer drugs, including IRI, Oxa and Taxol, compared to parental WiDr cells (Figure 1). In vivo, WiDr/UGCG^−^ tumors are sensitive to Oxa treatments, which is one of the first-line drugs used for colorectal cancer (Figure 5). This specific approach for targeting the UGCG gene immensely decreases the tumorigenesis and cell migration of WiDr/UGCG^−^ cells in tumor sphere formation, wound healing and tumor growth in mice (Figure 2 and Figure 5). Indeed, UGCG knockout eliminates the enrichment of colon CSCs in WiDr/UGCG^−^ cells and tumors (Figure 3 and Figure 5D). These effects of UGCG knockout on WiDr/UGCG^−^ cells are further supported by the evidence from treatments with Genz-161, a novel GCS inhibitor [6,42]. Lower doses of Genz-161, either in cell models (4 μM) or in tumor-bearing mice (4 mg/kg, i.p., per every 3 days), sufficiently reverse drug resistance and decrease tumor growth without significant adverse effects (Figure 1 and Figure 5A, Supplementary Figure S1). These results not only indicate the key role played by GCS in tumor progression, but also indicate that Genz-161 has a potential to be a potent therapeutic agent for cancers harboring homozygous p53 mutations, at least p53 R273H.

Suppressing ceramide glycosylation by GCS provides an effective option for targeting p53 mutant cancers. Targeting p53 mutations, particular missense mutations that are frequently detected in many types of cancer and promote tumor progression, is crucial for improving the outcome of cancer treatments [16,20,43,44,45]. Among these available options, restoring the expression of wild-type p53 protein, which can eliminate the dominant effects of missense mutant p53 proteins incorporated in the hetero-tetramers in cancer cells, might be more effective but faces a great challenge [11]. Intriguingly, our previous studies elucidated that inhibition of Cer-glycosylation restores functional p53 tumor suppression in heterozygous models (p53 R273H^−/+^) generated by knock-in editing [6,9,11]. Furthermore, the present study demonstrates that inhibition of GCS increases p53-associated tumor suppression in WiDr colon cancer cells carrying homozygous mutations (p53 R273H) (Figure 4 and Figure 7). Congruous with the effects of reversing drug resistance and tumorigenesis, UGCG knockout or the GCS inhibitor Genz-161 strikingly upregulated the protein levels of pp53 with p53-responsive genes (p21 and Bax) in homozygous p53-mutant WiDr cells, either in cell culture treated with IRI or in tumor-bearing mice treated with Oxa (Figure 4 and Figure 7).

The RNA m^6^A modification is involved in modulating the expression of p53 mutant proteins. The RNA m^6^A methylation that is mainly catalyzed by METTL3 in human cells can manipulate the fate of mRNA in producing protein in pre-mRNA splicing, mRNA stabilization and nuclear export for translation [9,46,47]. RNA m^6^A methylation and further modification with m^6^A readers, a group of proteins that bind to m^6^A-RNA, are correlated with the expression of p53 mutant and neoplastic transformation [9,48,49]. Enhanced GSLs, particular Gb3 in GSL-enriched microdomain, can increase the expression of METTL3 via cSrc and β-catenin signal transduction pathway [6,9]. Our previous studies showed that RNA m^6^A methylation by METTL3 at the mutant codon (R273H) regulated the expression of mutant protein and GOF by SW48/TP53 Dox cells (*TP53* R273^−/+^) [9]. In the current study, we found that drug-induced cell stress significantly enhanced METTL3 protein levels in WiDr cancer cells or tumors (Figure 4 and Figure 7B,C). Conversely, inhibition of GCS, using either UGCG knockout or Genz-161, markedly eliminated METTL3 protein expression in cell models and xenograft tumors (Figure 4 and Figure 7B,C). The inhibition of RNA m^6^A methylation using NPC (Figure 8A) inspiringly promoted the expression of pp53 and p53-responsive genes (p21, Bax) in WiDr cells carrying *TP53* R273H, rather than in SW48 cells (Figure 8C,D). NPC treatments significantly re-sensitized WiDr cell response to IRI (Figure 8B), further indicating that the inhibition of RNA m^6^A methylation can advance tumor suppression in p53 mutant status. The present study indicates that RNA m^6^A modification can modulate the expression of functional p53 protein(s) in cancer cells carrying the homozygous *TP53* R273 mutation, for example in colon cancer WiDr cells. It is possible that RNA-m^6^A modification modulates the processes, either pre-RNA splicing or protein phosphorylation, to produce modified mutant p53 proteins (mm p53) in cancer cells carrying mutations (Figure 9). Further studies in other mutant models and genetic manipulation of METTL3 or other enzymes involved in RNA m^6^A modification and protein phosphorylation would consequently uncover the mystery of the homozygous status.

Altogether, our present study demonstrated that Cer-glycosylation via RNA m^6^A methylation upregulates the expression of p53 R273H and its GOF in cancer cells in response to chemotherapy. Conversely, the suppression of Cer-glycosylation by a GCS inhibitor, or directly repressing m^6^A modification, can markedly increase p53-associated tumor-suppressive function and re-sensitize drug-resistant cancerous cells and tumors to chemotherapeutic agents. Modulating mutant protein expression in cancer cells, particularly in the homozygous status, is more sophisticated than our current understanding. Further studies in other mutant cancer models are highly needed. Supported with present findings, the inhibition of Cer-glycosylation by GCS emerges as an achievable therapeutic approach for targeting certain gene mutations to improve cancer treatments.

## 4. Materials and Methods

### 4.1. UGCG Knockout Cell Lines Development and Cell Culture

The human WiDr (missense mutation *TP53* R273H) colon cancer cell line was purchased from American Type Culture Collection (ATCC; Manassas, VA, USA) [6,50]. Its sublines, WiDr/UGCG^−^, were generated by knocking out human UGCG (NCBI gene ID: 7357) via CRISP/cas9 gene editing by Gene Script (Piscataway, NJ, USA). Briefly, target sites were located by a guidance RNA (gRNA) and transient transfection of RNP (GenScript CRISPR single-guide RNAs:Cas9). The endogenous UGCG gene was targeted and mutated, resulting in consequential reduction in the expression of GCS. A single isogenic knockout cell clone from the selected ones was identified by Sanger sequencing screening and defined as WiDr/UGCG^−^. Primers for UGCG gRNA T3 site (5′>3′): forward, AGAGTAGAGAAGCCAGCACGA; reverse, CTAGAACACAGCAGGTTCCCA. Cells of WiDr or WiDr/UGCG^−^ were cultured in ATCC-formulated EMEM containing 10% fetal bovine serum (FBS), 100 units/mL penicillin, 100 µg/mL streptomycin and 584 mg/L l-glutamine. SW48 (wild type *TP53*) colon cancer cells (from ATCC) were cultured in RPMI-1640 media containing 10% fetal bovine serum (FBS), 100 units/mL penicillin, 100 μg/mL streptomycin and 584 mg/L l-glutamine. All cells were maintained in an incubator humidified with 95% air and 5% CO_2_ at 37 °C.

### 4.2. Cell Viability Assay

Cell viability was assessed using the CellTiter-Glo luminescent cell viability assay kit (Promega, Madison, WI, USA), as described previously [11,12]. Briefly, cells (4000 cells/well) were grown in 96-well plates overnight and then switched to 5% FBS medium containing drugs for 72 h treatments. For combination treatment, cells were cultured in 10% FBS medium containing Genz-161 (4 μM) for 48 h in advance and placed into 96-well plates for overnight growth and co-cultured with drugs for an additional 72 h. Cell viability was assessed in a Synergy HT microplate reader (BioTek, Winnooski, VT, USA), following incubation with reagent of CellTiter-Glo Luminescent Cell Viability kit (Promega, Madison, WI, USA). A new GCS inhibitor, Genz-161 (GENZ 667161, (*S*)-quinuclidin-3-yl(2-(2-(4-fluorophenyl)thiazol-4-yl)propan-2-yl carbamate) was kindly provided by Sanofi Genzyme (Framingham, MA, USA) [51,52]. Oxaliplatin, paclitaxel and irinotecan hydrochloride were purchased from Sigma-Aldrich (St. Louis, MO, USA). Neplanocin A (a potential m^6^A methylation inhibitor, NPC) was purchased from Cayman Chemical (Cayman Chemical, Ann Arbor, MI, USA).

### 4.3. RT-qPCR Analysis

This assessment was performed as described previously [12,25]. Briefly, total RNA was extracted from cells or tissues using an SV Total RNA Isolation kit (Promega, Madison, WI, USA). Equal amounts of RNA (100 ng/reaction) of samples were applied for assays using OneStep RT-PCR kit (Qiagen, Germantown, MD, USA). Pairs of primers synthesized from IDT (Coralville, IA, USA) for UGCG (forward, 5′-CTTGGTTCACGGGCTGCCTTAC-3′; reverse, 5′-GAAACCAGTTACATTGGC AGAGAT-3′) were used in PCR amplification. The PCR amplification was performed in 35 cycles of denaturation at 94 °C for 30 s, annealing at 55 °C for 30 s, and extension at 72 °C for 60 s. Glyceraldehyde-3-phosphate dehydrogenase (GAPDH), an endogenous control (forward, 5′-GTCTCCTCTGACTTCAACAGCG-3′; reverse, 5′-ACCACCCTGTTGCTGTAGCCAA-3′) was amplified.

### 4.4. Tumor-Sphere Formation Assay

The assay was conducted as previously described with minor modification [8,53]. Briefly, cells of WiDr or WiDr/UGCG^−^ (passages 3–10; 500 cells) in 10% FBS EMEM medium were placed into 96-well ultra-low binding microplates and cultured for 6 days. Images of tumor spheres were captured using an EVOS FL cell imaging system with color CCD camera (100× magnification; Thermo Fisher Scientific, Waltham, MA, USA). Cell viability of tumor spheres was further assessed with CellTiter-Glo reagent, as described above (Cell viability assay).

### 4.5. Wound Healing Assay

The assay was conducted as described previously [11,54]. Briefly, cells (2 × 10^5^/well) were planted in 6-well microplates in 10% FBS EMEM medium with agents, and the wounds were scratched with 100-μL pipette tips after 24 h of growth (~80% confluency). The wounded areas were observed and captured using EVOS FL cell imaging system (100× magnification). For combination treatments, WiDr cells were pre-treated with Genz-161 (4 μM, 48 h) and then treated with IRI for an additional 48 h.

### 4.6. Imaging Flow Cytometry Analysis

Imaging flow cytometry was carried out as described previously [9,11]. After treatments, suspended cells (10^6^ cells/mL) were incubated with human CD44v6 Alexa Fluor^®^ 488-conjugated antibody (2F10; mouse IgG1; from R&D Systems, Minneapolis, MN, USA) and human CD133 APC-conjugated antibody (170411; mouse IgG2b; from R&D Systems) in 1% BSA-containing PBS at 4 °C for 45 min. After washing, cells were resuspended in 1% BSA PBS (5 × 10^5^ cells/150 μL) and analyzed using an Amnis Imagestream Mark II Imagestream INSPIRE software (v200.1), and the data were further analyzed using the IDEAS v6.2 program.

### 4.7. Western Blot Analysis

Western blotting was carried out as described previously [11,12,28]. Briefly, cells or tissue homogenates were lysed in NP40 cell lysis buffer containing protease inhibitors (Biosource, Camarillo, CA, USA) to extract total cellular proteins following these treatments. Equal amounts of proteins (50 µg/lane) were resolved by using 4–12% gradient SDS-PAGE (Life Technologies). The nitrocellulose-membrane blots transferred were blocked in the StartingBlock Blocking buffer (proprietary protein formulation in 0.05% Tween-20, 20 mM phosphate-buffered saline, pH 7.5 (PBST), Thermo Fisher Scientific), and then incubated with each one of the primary antibodies (1:1000 or 1:2000 dilution) at 4 °C overnight. These blots were incubated with corresponding horseradish peroxidase–conjugated secondary antibodies (1:5000 dilution) and detected using SuperSignal West Femto substrate (Thermo Fisher Scientific) and ChemiDoc MP imaging system (Bio-Rad, Hercules, CA, USA). Glyceraldehyde-3-phosphate dehydrogenase (GAPDH) was used as a loading control for cellular protein. Antibodies against human p53 phosphorylated at Ser15 were purchased from Cell Signaling Technology (Danvers, MA, USA). Mouse monoclonal antibody (IgG κ, 1E5) against human GCS protein (amino acids 33–131) was purchased from Santa Cruz Biotechnology (cat# sc-293235; Dallas, TX, USA). Antibodies for p21, Bax, p53, and GAPDH were obtained from Santa Cruz Biotechnology (Dallas, TX, USA). Relative protein levels were calculated from optical density values for each protein band using the Image Lab software v6.1 (Bio-Rad, Hercules, CA, USA), normalized against those for GAPDH from three separate blots.

### 4.8. Animal Studies in Tumor-Bearing Mice

All animal experiments were approved by the Institutional Animal Care and Use Committee, University of Louisiana at Monroe (ULM; protocol# 23MAR-YYL-01, approved 21 March 2023), and were carried out in strict accordance with good animal practice as defined by NIH guidelines. Athymic nude mice (Foxn1^nu^/Foxn1^+^, 7–9 weeks, female and males) were purchased from Envigo (Indianapolis, IN, USA) and maintained in the vivarium at ULM. Animal studies were conducted as described previously [11,12]. Briefly, cell suspension of WiDr/mock and WiDr/UGCG^−^ (5–7 passages, 2.5 × 10^6^ cells in 40 μL/each) was subcutaneously injected into the left flank or both left and right flanks of the mice. Mice with tumors (~5 mm in diameter) were randomly allotted to different groups (6 mice/group, including 3 males and 3 females) for treatments. For treatments, Oxaliplatin (Oxa, 2.0, 4.0 mg/kg, once every 6 days) or irinotecan (IRI, 6 mg/kg) was administered intraperitoneally alone or with Genz-161 (4.0 mg/kg once every 3 days, in mice bearing WiDr tumors). Mice were monitored by measuring tumor sizes, body weights, and clinical observations twice a week. Bone marrow samples were extracted and counted with hemocytometer as described previously [55]. Tumors and other tissues were dissected and stored at −80 °C for further analyses.

### 4.9. Immunohistochemistry

Immunocytochemistry assessments were performed as described previously [11,12,28]. Microsections (5 μm) of tumors were prepared and stained with hematoxylin and eosin (H&E) from AML Laboratories (St. Augustine, FL, USA) and further characterized by a pathologist. Antigens were retrieved in steaming sodium citrate buffers (10 nM, 0.05% Tween-20, pH 6.0). After blocking with 5% goat serum in PBS, slides were incubated with primary antibodies or conjugated antibodies (1:100) in blocking solution at 4 °C overnight. For the detection of colon CSCs, the blocked slides were incubated with CD44v6 Alexa-Fluor 488-conjugated antibody (2F10; mouse IgG1; purchased from R&D Systems, Minneapolis, MN, USA) and CD133/2 APC-conjugated antibody (293C3, mouse IgG2b; purchased from Miltenyi Biotec, San Diego, CA, USA) (1:100). Tumor ceramide and phosphorylated p53 (pp53) were recognized by antibodies against ceramide (clone MID 15B4, Millipore Sigma, St. Louis, MO, USA) and pp53 (Cell Signaling Technology; Danvers, MA, USA). Corresponding Alexa Fluor 488-conjugated anti-mouse IgG and Alexa Fluor 594-conjugated anti-rabbit IgG (1:1000) were applied for further incubation to recognize the corresponding primary antibodies. After washing, cell nuclei were counterstained with DAPI (4′,6-diamidino-2-phenylindole) in mounting solution (Vector laboratories, Burlingame, CA, USA). Images (200× magnification) were captured using the EVOS FL cell imaging system with a color CCD camera (Life Technologies, Grand Island, NY, USA). Alexa Fluor 488-conjugated anti-mouse IgG and Alexa Fluor 594-conjugated anti-rabbit IgG were purchased from Thermo Fisher Scientific. The numbers of colon CSCs (CD44v6^+^/CD133^+^) or the relative levels of ceramide or pp53 in tumor cells were analyzed against total tumor cells (DAPI^+^) using ImageJ program V0.6.0 (https://ij.imjoy.io/, accessed on 18 February 2026).

### 4.10. Shotgun Lipidomics Analysis

Mass spectrometry-based shotgun lipidomics analysis was conducted, as described previously [56,57]. Briefly, after treatments, tumor tissues were freshly dissected and stored at −80 °C (5 mg each). Tissue samples were further homogenized in 0.1× PBS. Protein concentrations were measured using a bicinchoninic acid (BCA) assay prior to lipid extraction. A premixture of lipid internal standards including N18:0-d35 GalCer (20 nmol/mg proteins) for cerebroside quantitation was separately added to individual homogenates based on the protein content of the sample. Lipid extraction was conducted by using a modified Bligh–Dyer method, as previously described [58]. Each lipid extract was reconstituted with a volume of 500 μL/mg protein in CHCl_3_/MeOH (1:1, *v*/*v*). The lipid extracts were finally flushed with nitrogen, capped, and stored at −20 °C for ESI/MS analyses.

ESI/MS quantitative analyses of cerebroside species were achieved using a triple–quadrupole mass spectrometer (TSQ Altis^TM^ mass spectrometer, Thermo Fisher Scientific, Waltham, MA, USA) coupled with a Nanomate device (Advion, Ithaca, NY, USA) and controlled by the Xcalibur system [59]. Major cerebroside molecular species were directly quantitated by comparing ion peak intensity with that of an internal standard (i.e., N18:0-d35 GalCer) from the first-dimensional mass spectrum after correction for the different ^13^C-isotopologue intensities of cerebroside species relative to the internal standard in the positive-ion mode. By using these previously quantified major cerebroside species as a set of standards in addition to the original internal standard (i.e., N18:0-d35 GalCer), minor cerebroside species were quantitated/refined by MS/MS in a 2D mass spectrometric manner [36]. Data processing, including ion peak selection, baseline correction, data transfer, peak intensity comparison ^13^C deisotoping and quantitation, was facilitated by a custom-programmed Microsoft Excel macro as previously described [60,61], ensuring accurate analysis of lipid molecular species. Quantitative data from biological samples were normalized to the protein content of the tissues, and all data are presented as means ± SD of samples.

### 4.11. ELISA of m^6^A-RNA

The amounts of m^6^A-RNA were analyzed using the EpiQuick m^6^A RNA methylation quantification kit (colorimetric; EpigenTek^TM^, Famingdale, NY, USA), according to the manufacturer’s instruction [62]. Briefly, total RNA was extracted from cells treated with NPC (200 nM, 48 h) using SV total RNA isolation kits (Promega, Madison, WI, USA). Extracted RNA (200 ng/reaction) of each sample were utilized in the ELISA assay and quantitated with the m^6^A standard curves.

### 4.12. Data Analysis

All experiments in cell models were performed in triplicate and repeated twice unless otherwise stated. Data are expressed as mean ± SD. One-way or two-way ANOVA was used for multiple group comparisons, as appropriate, followed by Tukey’s multiple comparisons test. Statistical analyses were performed using Prism v10 (GraphPad, San Diego, CA, USA). A *p*-value < 0.05 was considered statistically significant.

## Data Availability

The original contributions presented in this study are included in the article and Supplementary Materials. Further inquiries can be directed to the corresponding authors.

## Supplementary Materials

The following supporting information can be downloaded at https://www.mdpi.com/article/10.3390/ijms27073237/s1.
