# Supplementary material for: Suppression of Glucosylceramide Synthase Reverses Drug Resistance in Cancer Cells Harboring Homozygous p53 Mutants
Source: Int J Mol Sci. 2026 Apr 2;27(7):3237. doi: 10.3390/ijms27073237 (PMC13073438; doi:10.3390/ijms27073237)
Supplement: Supplementary file 1 [file ijms-27-03237-s001.zip › ijms-4180081-supplementary.pdf]

Supplementary Figures

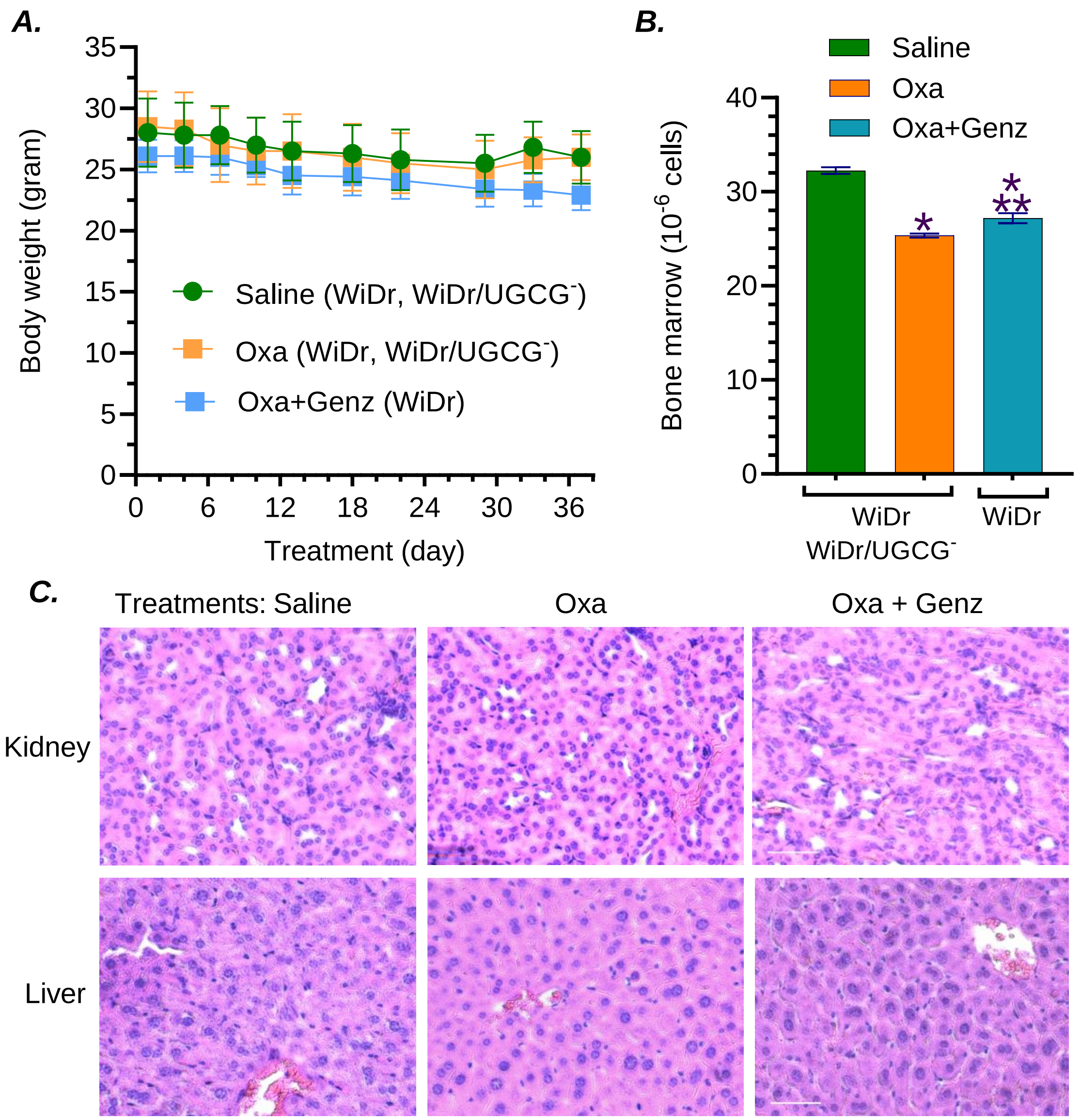

Figure S1. Tumor-bearing mice were treated with Oxa alone or combined with Genz-161. Mice bearing tumors (left frank, WiDr; right frank WiDr/UGCG-) were treated with saline or oxaliplatin (2 mg/kg, ip, every 6 days; 6 mice/group, m/f). Mice bearing WiDr tumors were treated with combination (oxaliplatin 2 mg, ip, every 6 days plus Genz-161 4 mg/kg, ip, every 3 days; 6 mice/group, m/f). ***A***, Body weights. ***B***, Bone marrow. *, *p*<0.001 compared with mice treated with saline. **, *p*<0.001 compared with mice treated with oxaliplatin. ***C***, Kidney and liver sections stained with H&E (200x magnification). Scale bar represents to 50 μm..

Mass Spectrometry-based Shotgun Lipidomics

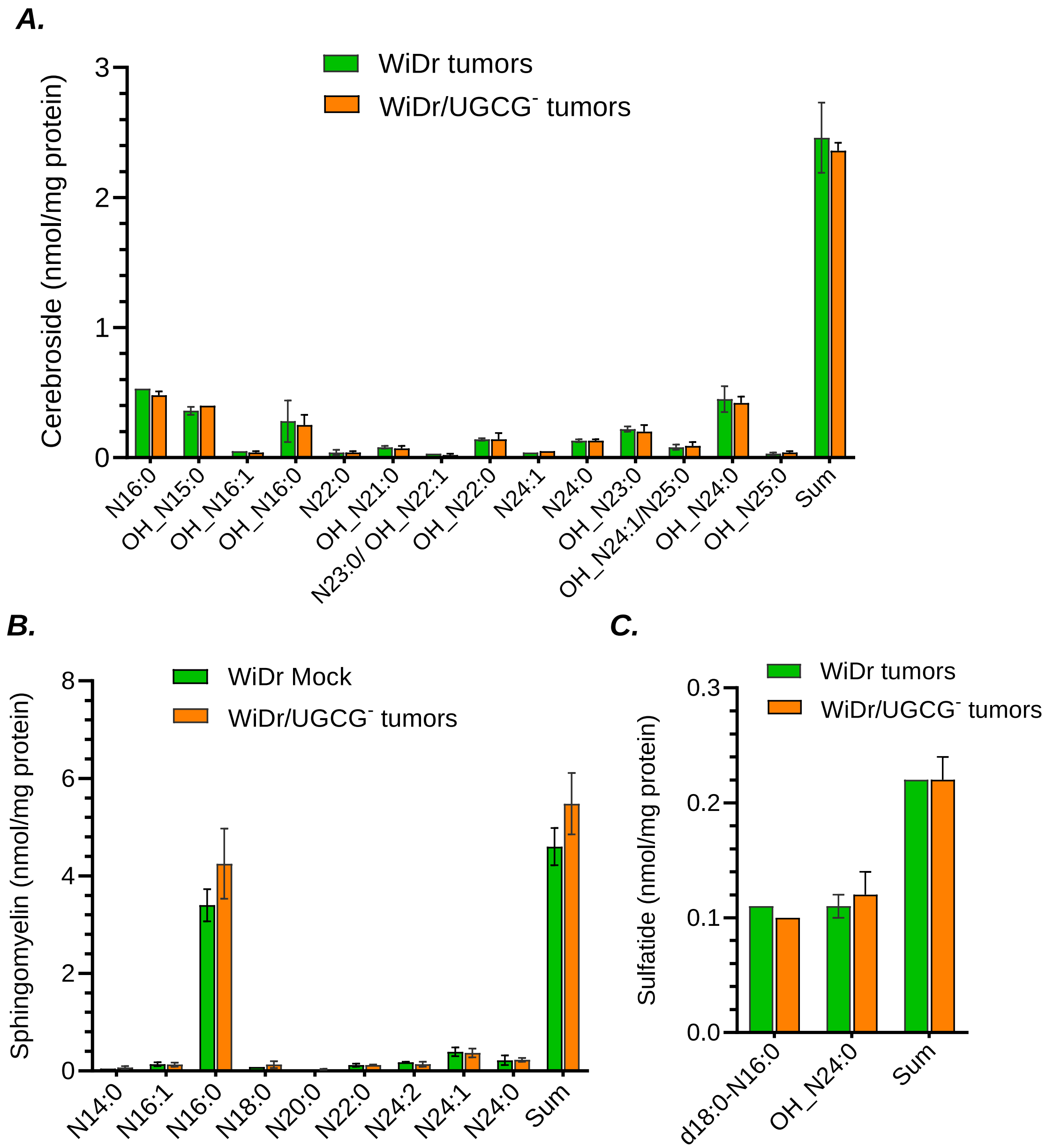

Figure S2. Mass Spectrometry-based Shotgun Lipidomics. Tumor-bearing mice were treated with Oxa (2 mg/kg, i.p, once every 6 days) for 37 days. ***A***, Cerebroside of tumors treated with Oxa. ***B***, Sphingomyelin of tumors treated with Oxa. ***C***, Sulfatide of tumors treated with Oxa.
